# A Retrospective Analysis of Oxygen Desaturation during Acoustic Respiratory Rate Monitoring in Non-ICU Patients following Tracheal Extubation after General Anesthesia

**DOI:** 10.1155/2017/4203156

**Published:** 2017-04-12

**Authors:** Hideaki Kawanishi, Satoki Inoue, Masahiko Kawaguchi

**Affiliations:** Department of Anesthesiology and Division of Intensive Care, Nara Medical University, 840 Shijo-cho, Kashihara, Nara 634-8522, Japan

## Abstract

*Purpose*. Acoustic respiratory rate (RRa) monitoring provides an accurate estimation of the respiratory rate (RR). We investigated the incidence of oxygen desaturation under RRa monitoring in a postoperative setting and identified its related factors.* Methods*. This study was a retrospective chart review of postoperative patients outside an intensive care unit setting. Using the data collected during the first 8 h postoperatively, patients were divided into oxygen desaturated (SpO_2_ < 90% for >10 s) and nondesaturated groups under oxygen administration. Multivariate analysis was used to determine oxygen desaturation-associated explanatory factors.* Results*. Oxygen desaturation was detected in 102 of 935 patients (10.9%). % vital capacity [odds ratio (OR), 0.885 per 10% increase; 95% confidence interval (CI), 0.790 to 0.992], coexisting chronic obstructive pulmonary disease (OR, 2.195; 95% CI, 1.088 to 4.428), and absence of a critical RRa change (RR > 30 or <8 beats/min for >2 min) (OR, 1.972; 95% CI, 1.226 to 3.172) were independently associated with oxygen desaturation.* Conclusion*. Postoperative oxygen desaturation was observed in more than 10% of the patients whose RR was monitored by RRa under oxygen therapy. It is more likely to occur in patients with impaired pulmonary function or morbid pulmonary status and can also occur in the absence of abnormal RR.

## 1. Introduction

There is an increasing recognition of the importance of continuous patient monitoring under various clinical conditions. Pulse oximetry (SpO_2_) allows for a continuous, noninvasive monitoring of oxygenation; however, supplemental oxygen may delay the diagnosis of hypoventilation caused by bradypnea or respiratory deterioration [1, 2]. Changes in respiratory rate (RR) require intensive care unit admission [3], and a continuous monitoring of the heart rate and RR in the medical-surgical unit reduces the duration of hospital and intensive care unit stay and code-blue rates [4]. Thus, the Anesthesia Patient Safety Foundation recommends both SpO_2_ and respiration rate monitoring, particularly after extubation and narcotic analgesic use for pain management [2, 5]. Noninvasive respiratory monitoring device using adhesive sensors with an integrated acoustic transducer positioned on the patient's throat [Rainbow Acoustic Monitoring™ (RAM), Masimo Corp., Irvine, CA, USA] is a relatively new method of ventilation monitoring, and it provides an accurate estimation of RR in the postanesthesia care unit [6, 7], intensive care unit [8], and emergency room [9].

As described above, SpO_2_ alone is not sufficient to detect hypoventilation in patients receiving supplemental oxygen [1, 2], which may suggest that oxygen saturation is usually maintained even with hypoventilation for postoperative patients receiving supplemental oxygen. Therefore, the average postoperative patients receiving supplemental oxygen may rarely develop hypoxia provided that normoventilation is maintained. Based on this theory, we have implemented the use of a pulse oximeter with an acoustic RR (RRa) monitoring device (Rad-87™, version 7805, Masimo Corp.) in postoperative settings outside the intensive care unit. This method has been implemented to prevent the development of oxygen desaturation, as caregivers generally intervene with an RR alert. However, we have occasionally encountered desaturated patients under this monitoring system. In the current study, we investigated the incidence of oxygen desaturation under RRa monitoring in a postoperative setting and identified factors related to its development.

## 2. Methods

Approval for access to the data stored on the RRa device pulse oximeter and the review of clinical charts of postoperative patients with continuously monitored RRa was obtained from the Nara Medical University Institutional Review Board (number 480-3 approved on 04-14-2014), and written informed consent for this historical study was waived.

### 2.1. Standard Perioperative Patient Treatment

Cases requiring intensive care or similar treatment were excluded from RRa monitoring since mechanical ventilation or manual RR monitoring, such as intermittent auscultation every 1-2 h, was required for these cases. RRa monitoring was applied to extubated surgical patients who were outside of the intensive care units. The application was limited to elective cases, partially due to a shortage of RRa devices. In fact, patients who underwent craniotomy, thyroidectomy, spinal surgery, laparotomy, laparoscopic surgery, and major orthopedic surgery were initially considered as candidates for RRa monitoring.

The methods of anesthetic induction and maintenance, as well as tracheal intubation, were not standardized for each patient. Anesthesia was maintained with sevoflurane or propofol, and fentanyl or remifentanil was used for analgesia. Rocuronium was used for neuromuscular blockade and sugammadex for the reversal of the neuromuscular blockade after evaluating the status of the neuromuscular blockade using a nerve stimulator. Fluid management was performed at the discretion of the attending anesthetist and a transfusion was performed if necessary. Postoperative analgesia was provided with intravenous fentanyl (10–40 *μ*g/h) or epidural ropivacaine (0.1–0.2%, 2–4 mL/h) combined with fentanyl (10–20 *μ*g/h) using a patient-controlled analgesia (PCA) device (Coopdech Syrinjector PCA DeviceTM for iv, Coopdech Balloonjector PCA DeviceTM for epidural, Daiken Medical Co. Ltd., Osaka City, Osaka, Japan). The PCA bolus sizes and lockout timings were set at 10–40 *μ*g/h and 10 min for iv and 3 mL and 30 min for epidural, respectively. When PCA was used, a low dose droperidol (1.25–2.5 mg/day) was combined with a PCA device. Upon discharge from the operating room, an adhesive acoustic respiration sensor (RAS-125™ or RAS-125™ rev C, Masimo Corp.) and an oximetry sensor (LNCS Adtx, Masimo Corp.) were placed on the patient's neck and finger, respectively, and connected to an RRa device pulse oximeter to obtain the RR, SpO_2_, pulse rate (PR), and perfusion index (PI) measurements. PI with a trending capability reflects the arterial pulse signal strength and may be used as a diagnostic tool during low perfusion [10]. A low PI is associated with an increased discrepancy between SpO_2_ and arterial oxygen saturation (SaO_2_) [11].

According to the manufacturer's instructions, the acoustic sensor was placed on either side of the larynx, above the thyroid cartilage, and below the jaw line. Patients were then transferred to a general surgical ward. A general hospital setting had a nurse to patient ratio of 1 : 7 and was comprised of a surgical population undergoing miscellaneous surgeries with opioid-based, patient-controlled analgesia. In the case of an RRa or SpO_2_ alarm, the nurses at our institute were trained to deliver standard care and to pat a patient on the shoulder or chest while calling or orally encourage them to breathe deeply. However, the staff seldom adjusted the oxygen delivery rate. Patients were continuously monitored with an RRa device containing a pulse oximeter as per the standard of care, and the monitored data were stored in the internal memory. Oxygen (3–5 L/min) was administered for up to 8 h regardless of the postoperative analgesia used through an oxygen mask according to our institutional standard practice. RR, SpO_2_, and PR alarms were set at RR < 8 or >30 breaths per min, <90% SpO_2_ with supplemental oxygen, and PR > 130 or <50 beats per min, respectively, according to the institutional protocol. The RRa device pulse oximetry data were temporarily and automatically stored in an internal memory for up to 72 h per 2 s resolution. Every time a Rad-87 monitoring was terminated, the data were transferred to a storage device using downloaded software (TrendCom version 3460, Masimo Corp.) and were saved at our institute.

### 2.2. Data Handling

**Figure 1:**
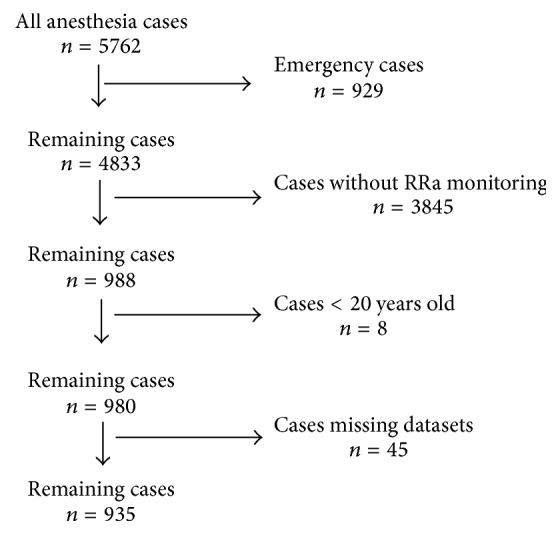
Study workflow.

Data were collected from May 1, 2012, to July 31, 2013. During this period, out of the 5762 anesthesia cases, 988 were monitored with an RRa device pulse oximeter. The cumulatively stored data were extracted, and those collected during the first preoperative 8 h were used for analysis. “Invalid” was marked on the artifactual periods in the stored data by sophisticated artifact detection algorithms. After prescreening the raw data, data reliability was confirmed. The exclusion criteria for the current study and consequent reductions in the eligible patients were as follows: (1) emergency cases (*n* = 929); (2) cases without RRa monitoring (*n* = 3845); (3) cases < 20 years old (*n* = 8); and (4) cases with missing datasets or signal loss during the recording process (*n* = 45). Finally, data from 935 patients were used in this study (Figure 1).

Perioperative data were extracted from the patients' clinical charts. Based on the analyzed data, an event of oxygen desaturation was defined by a SpO_2_ < 90% for 10 s or more, which was an arbitrary local rule, during 8 h of recording. To assess the data reliability, PI data were also extracted. Subsequently, the patients were divided into the following two groups: (1) the noncritical RR change group and (2) the critical RR change group. Critical RR change was defined as an RR < 8 or >30 breaths per min in 2 min or longer. Initially, we expected to prevent oxygen desaturation in advance by intervening when an abnormal RR was observed. However, upon implementation of the caregiver treatment under RRa monitoring, we found that it took at least 2 min for the caregivers to intervene after the RR alarm went off.

### 2.3. Statistical Analysis

Univariate analysis was used to identify factors associated with oxygen desaturation. Explanatory factors having a significant univariate association (*P* < 0.15) with oxygen desaturation were used to perform multivariable logistic regression analysis and stepwise backward elimination. All candidate variables were entered in the initial model and at each step; the variable with least significance (i.e., the highest *P* value of >0.15) was eliminated and presented as adjusted odds ratios with 95% confidential intervals (CI). Interactions between variables were systematically searched and collinearity was considered for *r* or rho > 0.8 using Pearson or Spearman coefficient matrix correlation, respectively. Discrimination of the final model for oxygen desaturation was assessed using the likelihood ratio test. Calibration of the model was tested using the Hosmer-Lemeshow statistic. The area under the receiver operating characteristic (ROC) curve was computed as a descriptive tool for measuring model bias. Data were expressed as mean (SD) for normal distribution and as median (IQR) for a non-Gaussian distribution. Comparison of two means was performed using Student's *t*-test and that of two medians and two proportions was performed using the Mann–Whitney *U* test and *χ*^2^ test or Fisher's exact method, respectively. The MedCalc statistical package (version 14.12.0, MedCalc Software BVBA, Ostend, Belgium) was used to perform all the analyses.

## 3. Results

No patients died or required a code-blue call or intensive care during the study period, and there were no reports of any critical complication. To investigate the overall incidence of oxygen desaturation, we used data from 935 patients with complete datasets and without signal loss during recording. Oxygen desaturation was observed in 102 (10.9%) patients. The median number of oxygen desaturation events was two (range 1–34). The median of the lowest SpO_2_ level was 84% (range 36%–89%) and the median of the longest duration was 48 s (range 10–390 s). Patient data and perioperative characteristics were compared between the desaturation and nondesaturation groups (Table 1). Univariate analysis revealed that coexisting COPD (*P* = 0.001), low % VC (*P* = 0.045) and FEV_1_% (*P* = 0.010), long duration of anesthesia (*P* = 0.026) and surgery (*P* = 0.027), low PI (*P* = 0.028), large fluid balance (*P* = 0.021), transfusion requirement (*P* = 0.013), and the lack of a critical RRa change (*P* = 0.005) were all associated with oxygen desaturation.

Multivariate logistic regression analysis was performed, with intraoperative fluid balance categorized into quartile intervals, assuming that the risk increased in a linear fashion among the categories as data obtained from variables were not assumed to be normally distributed. Multivariate analysis revealed that % VC [odds ratio (OR) 0.885 per 10% increase; 95% confidence interval (CI), 0.790 to 0.992], coexisting COPD (OR, 2.195; 95% CI, 1.088 to 4.428), and absence of a critical RRa change (OR, 1.972; 95% CI, 1.226 to 3.172) were independently associated with oxygen desaturation (Table 2). Discrimination of the final model assessed by the likelihood ratio test was significant (*P* < 0.0001). Furthermore, the Hosmer-Lemeshow statistic did not reject a logistic regression model fit (*P* = 0.888). The explanatory model based on these variables had an area under the ROC curve of 0.661 (95% CI, 0.630 to 0.692).

Post hoc power calculations were performed for these stepwise backward multivariate logistic regression models using eight variables in the model. We followed standard methods to estimate the sample size for multivariate logistic regression, with at least ten outcomes needed for each included independent variable [12]. With a 10.9% (102/935) incidence of desaturation in the overall study population, we required 733 patients monitored by the RRa device pulse oximeter to appropriately perform multivariate logistic regression with eight variables. This demonstrates that our sample size was sufficient to build the model.

## 4. Discussion

Desaturation under oxygen administration was observed in 10.9% of the overall study population whose RR was monitored using RRa devices. Regarding causative factors for postoperative oxygen desaturation, study results showed that coexisting COPD and preoperative low % VC may increase the possibility of developing postoperative oxygen desaturation. Interestingly, this analysis also revealed an independent association between the absence of a critical RR change and desaturation under oxygen administration.

The rate of postoperative oxygen desaturation has been reported to vary, for example, from 20% to 50%, although comparison with the results of other studies remains limited due to varying definitions of oxygen desaturation [13, 14]. In addition, previous reports were not always based on the observation of the environment being left in supplemental oxygen. However, it has been reported that only less than 1% of the patients developed oxygen desaturation (SpO_2_ < 90%) when oxygen was administered [13]. Our study rate of 10.9% seems high for settings under supplemental oxygen. Compared with the short postoperative transport period [13], a relatively long observation period in our study likely increased the number of patients developing oxygen desaturation. Factors contributing to postoperative oxygen desaturation have been investigated, and it has been reported that the most important predictor of oxygen desaturation in the postoperative settings is patient care without oxygen supplementation [13]. In our study, all postoperative patients were given oxygen. In other studies, preexisting comorbidities have been reported as contributing factors for postoperative oxygen desaturation. Although low % VC has not been reported, smoking and a positive history of pulmonary disease have also been identified as contributing factors [14, 15]. Therefore, coexisting COPD and preoperative low % VC can be considered as the contributing factors for postoperative oxygen desaturation, even with supplemental oxygen, although identification of these factors is not novel.

The reason for the phenomenon that the incidence of desaturation predominantly occurred in patients without a critical RR change may be due to the effectiveness of the caregiver treatment under RRa monitoring. Such individuals could have reduced the postoperative oxygen desaturation primarily in cases with a critical RR change because the absence of a critical RR change generally suggests a stable respiratory status. Therefore, despite being described as the “neglected vital sign,” continuous RR monitoring may provide additional benefits for detecting hypoventilation before desaturation under oxygen administration [16]. However, it might not be applicable in patients with abnormal conditions, such as patients with impaired pulmonary function or morbid pulmonary conditions, as shown by our multivariate logistic analysis and previous reports [14, 15]. In our study, the absence of an abnormal RR did not guarantee that such patients did not develop desaturation under our monitoring setting. It has been established that respiratory rate is a useful vital sign and parameter for postoperative patient monitoring. Thus, we need to recognize that our postoperative monitoring system is weak in preventing desaturation unrelated to breathing patterns. From a different perspective, it is possible that our postoperative management protocol had limitations. For example, narrow alarm ranges might increase nuisance false alarms, resulting in alarm fatigue [17]; however, a narrow resetting of the critical RR range could have improved the ability of RRa monitoring to predict an impending oxygen desaturation at an earlier stage in a susceptible population.

Our study has several limitations. First, due to the retrospective nature of the study design, there is a possibility that we have missed the important confounding factors related to oxygen desaturation. Second, acoustic waveforms obtained for patients showing a critical RR change were not confirmed by simultaneous listening of breathing sounds from the acoustic signal because specific software (TagEditor, Masimo Corp.) was necessary for the analysis. Therefore, some of the patients excluded at prescreening may have showed a false positive RR change. In contrast, recording or reading errors in normal acoustic waveforms are rare. A false negative RR change by RRa likely did not significantly affect our results. Another potential limitation of the study is that critical RR changes or oxygen desaturation might have occurred during the artifact periods detected by the Masimo algorithms. However, a system status alert would have made the nurses aware of the problem and likely did not alter the results of the study. Lastly, opioids used for postoperative management may have affected oxygen desaturation during oxygen administration. Our data suggest that the dosage of opioids was not different between the desaturated and nondesaturated groups. Generally, opioid overdose can cause bradypnea. Thus, we assume that the use of postoperative opioids in this study had little effect on the development of oxygen desaturation without a critical RR change.

In conclusion, postoperative desaturation under oxygen therapy was observed in more than 10% of the patients whose RR was monitored using RRa devices and is more likely to occur in patients with impaired pulmonary function or morbid pulmonary status. Interestingly, postoperative oxygen desaturation can be associated with the absence of abnormal RR, indicating that the caregiver treatment under RRa monitoring was effective. However, we need to know that standard oxygenation and ventilation monitoring is not sufficient for a certain postoperative population, although close monitoring like in intensive care units may not be necessary.

## Figures and Tables

**Table 1:** Demographic and clinical Characteristics for patients with desaturation and nondesaturation.

Variables	Desaturation (*n* = 102)	Nondesaturation (*n* = 833)	*P* value
Age (year)	63.3 (15.6)	62.5 (14.7)	0.607
Gender (M/F)	55/47	466/367	0.698
Height (cm)	160.4 (9.9)	160.7 (9.4)	0.778
Weight (kg)	58.3 (11.7)	59.0 (12.1)	0.588
Body mass index (kg m^−2^)	22.6 (3.5)	22.8 (3.7)	0.632
% VC (%)	101.0 (19.0)	104.9 (18.3)	0.045
FEV_1.0_ (%)	74.8 (11.1)	77.9 (11.5)	0.010
Pulmonary dysfunction (Y/N)	117/242	175/401	0.478
ASA physical status (I/II/III)	13/79/10	146/631/56	0.290
Hypertension (Y/N)	47/55	347/486	0.393
Ischemic heart disease (Y/N)	7/95	36/797	0.248
CHF (Y/N)	0/102	6/827	0.390
HD (Y/N)	3/99	18/815	0.616
Asthma (Y/N)	3/99	27/806	0.871
COPD (Y/N)	16/86	54/779	0.001
DM (Y/N)	18/84	151/682	0.905

Duration of surgery (min)	276 (153)	244 (131)	0.027
Duration of anesthesia (min)	347 (162)	314 (138)	0.026

Surgical site			
Head and neck	26	257	0.208
Upper abdominal	12	109	
Lower abdominal	42	247	
Laparoscopy	18	171	
Other	4	49	

Transfusion (Y/N)	24/78	118/715	0.013
Total fluid balance (mL)	1785 (1118 to 2866)	1620 (978 to 2333)	0.021

Anesthetics			
Inhalational	25	626	0.940
Intravenous	77	207	

Rocuronium (mg)	95 (66 to 110)	90 (50 to 120)	0.612
Remifentanil (*μ*g)	1548 (731 to 2594)	1457 (652 to 2694)	0.440
Fentanyl (*μ*g)	300 (200 to 500)	300 (200 to 450)	0.976
PCA (Y/N)	70/32	523/310	0.248
Basal fentanyl dose by PCA (*μ*g/hr)	14.0 (0–55)	14.0 (0–45)	0.437

Critical RRa change (Y/N)	26/76	333/500	0.005

Mean perfusion index	2.09 (2.21)	2.51 (1.75)	0.028

Variables are expressed as mean (SD), median (IQR), or number.

FEV1%: forced expiratory volume 1.0 (sec) %, % VC: % vital capacity, pulmonary dysfunction: a case that had FEV1% < 70 or % VC < 80, ASA: American Society of Anesthesiologists, CHF: history of congestive heart failure, HD: on hemodialysis, COPD: chronic obstructive pulmonary disease, DM: diabetes mellitus, and surgical site: “upper abdominal” means supraumbilical procedures, “lower abdominal” means infra-abdominal procedures, and “laparoscopy” means any laparoscopic procedures. Rocuronium, remifentanil, and fentanyl: total doses of these drugs used intraoperatively, PCA: patient controlled analgesia, basal fentanyl dose by PCA: basal dose of fentanyl continuous administration delivered by a PCA device, and RRa: acoustic respiratory rate.

**Table 2:** Multivariate logistic regression model for desaturation.

	Odds ratio	95% CI	*P* value
% VC per 10%	0.885	0.790 to 0.992	0.036
FEV1% per 10%	0.861	0.705 to 1.051	0.140
COPD	2.195	1.088 to 4.428	0.028
Lack of RRa change	1.972	1.226 to 3.172	0.005
Mean perfusion index	0.890	0.781 to 1.014	0.079

% VC: % vital capacity, FEV1%: forced expiratory volume 1.0 (sec) %, COPD: chronic obstructive pulmonary disease, and RRa: acoustic respiratory rate.

Total fluid balance, transfusion requirement, and duration of surgery were excluded from the model during the stepwise backward elimination process.
